# Out-of-Hospital Care of Heart Failure Patients During and After COVID-19 Pandemic: Time for Telemedicine?

**DOI:** 10.3389/fdgth.2020.593885

**Published:** 2020-11-12

**Authors:** Alessandro Faragli, Edoardo La Porta, Carlo Campana, Burkert Pieske, Sebastian Kelle, Friedrich Koehler, Alessio Alogna

**Affiliations:** ^1^Department of Internal Medicine and Cardiology, Deutsches Herzzentrum Berlin, Berlin, Germany; ^2^Charité–Universitätsmedizin Berlin, Department of Internal Medicine and Cardiology, Campus Virchow-Klinikum, Berlin, Germany; ^3^Berlin Institute of Health (BIH), Berlin, Germany; ^4^DZHK (German Center for Cardiovascular Research), Partner Site Berlin, Berlin, Germany; ^5^Department of Cardionephrology, Clinical Ligurian Institute of High Specialty, Villa Maria Group (GVM) Care and Research, Rapallo, Italy; ^6^Department of Internal Medicine, University of Genoa and IRCSS Azienda Ospedaliera Universitaria San Martino-IST, Genoa, Italy; ^7^Unit of Dialysis, IRCSS Istituto Giannina Gaslini, Ospedale Pediatrico, Genoa, Italy; ^8^Department of Cardiology, Sant'Anna Hospital, ASST-Lariana, Como, Italy; ^9^Center for Cardiovascular Telemedicine, Department of Cardiology and Angiology, Charité–Universitätsmedizin Berlin, Berlin, Germany

**Keywords:** heart failure, COVID-19, home monitoring, body fluids, telemedicine

## Opinion Letter

The current letter has been driven by the clinical observation of the events that happened in the last months during the coronavirus disease 2019 (COVID-19) pandemic in the European countries, with specific reference to the situation of patients in the North of Italy and in Germany.

“*Specialists are people who always repeat the same mistake.”*—Walter Gropius, German architect and founder of the Bauhaus School

A 71-year-old male, Caucasian, is affected by chronic heart failure (CHF) New York Heart Association (NYHA) class III and chronic kidney disease stage III. The first diagnosis of CHF has been performed 4 years ago after hospitalization for acute coronary syndrome resulting in a percutaneous coronary intervention with primary stenting. Since then, he has been hospitalized at an average of 1.5 times per year. Two thirds of the patient's hospitalizations were caused by worsening of his chronic body fluid congestion with peripheral edema and impaired renal function, while for one third of the cases, the main cause was volume depletion. This has been manifesting with hypotension and hypokalemia as a result of challenges in managing the correct intake of diuretics and blood pressure-lowering medications.

When admitted to the cardiology ward, such a paradigmatic patient represents a challenge, especially with regard to the body fluid management. This requires a specialized heart failure (HF) team with extensive experience in the field. The clinical approach to such complex patients includes daily physical exam and control of body fluid balance through fluid intake and urine output. On top of this, biomarkers, chest X-ray, lung ultrasound, and, for cardiorenal patients, bioimpedance analysis are performed during the hospitalization to assess the patients' congestion status. Moreover, it requires a fine tuning of medications, diet, and liquid restrictions to achieve a proper balance between body volume and blood pressure.

After recompensation and discharge, the patient is left alone with a single method to monitor himself: a standard weight scale. He weighs himself every day, trying to keep contact with his physician on the phone. He relies on elective appointments in the outpatient clinic, three times a year.

It is February 2020, and the COVID-19 pandemic takes hold. The patient is not able to get a prompt appointment with his physician. In case of worsening of his clinical condition, he is being told to call the emergency number.

This situation could evolve into three different scenarios:

- No Hospitalization Needed and no SARS-CoV-19 Infection During the Pandemic

The patient independently manages his chronic fluid congestion based on the experience of the past years. He can avoid any contact with COVID-19^+^ patients. However, the lack of a proper medical assistance may increase the risk of experiencing a sudden decompensation event. Compared to the time pre-COVID-19 pandemic, his mortality risk may look the same in the short term, but it will probably drastically increase in the medium to long term.

- Clinical Deterioration of the Patient Condition

The patient constantly deteriorates, gains weight, and his quality of life is strongly affected. He does not get an appointment with his general practitioner, neither in the outpatient clinics. He manages to survive without an emergency hospitalization, but in a poor condition, for a few weeks or months until the pandemic situation has improved, and he receives medical attention.

- Hospitalization for Acute Decompensation

The patient gets admitted to the hospital because of an acute exacerbation of his condition. The hospitals are under great pressure because of the pandemic, and the intensive care units have limited capacity. His mortality risk may still be higher than before the pandemic.

- Hospitalization for COVID-19

The patient is infected with SARS-CoV-19, his condition quickly worsens, and he needs to be quickly hospitalized. The mortality risk in this scenario is possibly the highest.

The last two scenarios are unfortunate and, most importantly, avoidable. However, a drastic change in the management of chronic patients is today, more than ever, of paramount importance. In addition to the direct impact on public health, COVID-19 has been challenging the way of living, the habits, as well as many long-existing cultural and social structures on which societies are based. Maybe for the first time in modern medicine, the major strategy of healthcare policymakers has been to keep patients outside hospitals to avoid the spread of the infection. However, this is not enough, and remote monitoring strategies are necessary for the future of a sustainable healthcare system (1) for many reasons.

We are experiencing since many years a clear mismatch between specialized physicians and patients in need of care (2, 3). Aging in western countries has led to an increase in the number of patients with multiple comorbidities (4). Since the very first beginning of medicine and then throughout modern times, healthcare systems have been structured on a face-to-face patient–physician interaction. This kind of approach has contributed to a hospital influx of patients during the COVID-19 outbreak.

Several European healthcare systems seemed unprepared to fight the pandemic, while many hospitals even contributed to the initial spread of COVID-19. In this scenario, most of the scheduled medical and surgical procedures were rescheduled, while many chronic patients have been temporarily lost at follow-up.

For these reasons, telemedicine has turned from being a “nice to have” approach to an essential requirement (5) for a more efficient system. Chronic HF patients are facing an increased challenge regarding the management of body fluids.

**Figure 1:**
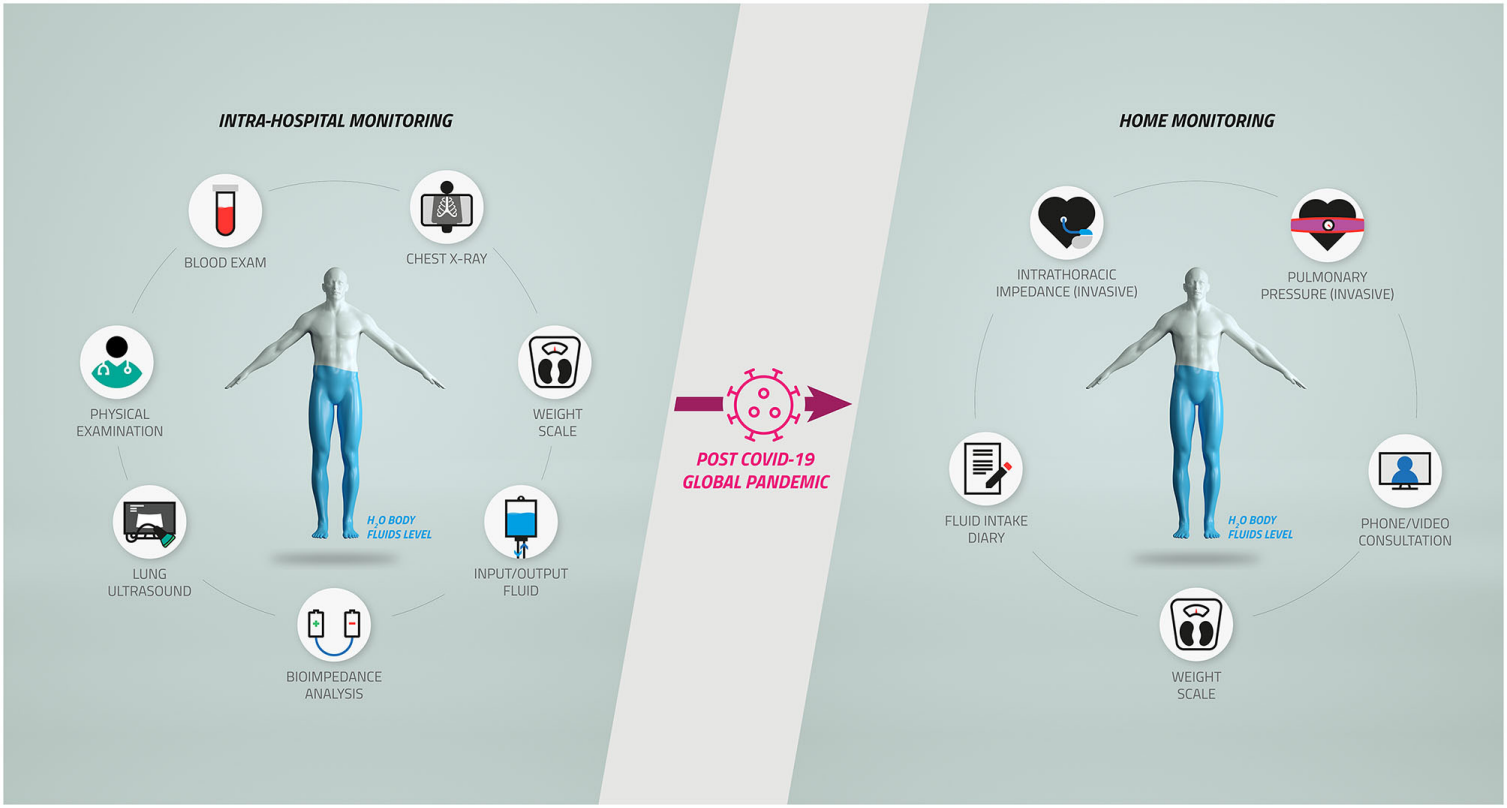
Body fluid management in chronic heart failure and cardiorenal patients after the coronavirus disease 2019 (COVID-19) pandemic. The complex workflow for the diagnosis and monitoring of body fluids of chronic heart failure and cardiorenal patients inside the hospitals **(Left)** has been restricted by the COVID-19 pandemic, causing a potentially relevant shift toward home monitoring protocols **(Right)**.

While during a hospitalization, the volume status of the patient is generally addressed by the medical doctors with various methods and solutions such as physical examination, ultrasound, chest X-ray, or blood examinations, at home, the solutions available and specific for the prevention of decompensation events in such patients are relatively limited, as described in Figure 1. We now believe that a natural shift toward home monitoring solutions should be considered and encouraged to manage the patients' body volume during and after the pandemic.

While invasive solutions such as cardioMEMS have already demonstrated to decrease HF patients' hospitalization (6), their utilization has been limited mainly due to invasiveness and related adverse events (7) or lack of penetrance among medical doctors (8). The spectrum of non-invasive telemedicine is, instead, broad, and the recent positive results achieved by Köhler et al. (9) and confirmed by the meta-analysis of Zhu et al. (10) are encouraging.

Audio–video tools able to connect patients and physicians for real-time consultations are widely available and, even if still not extensively adopted, during the pandemic, and the relative lockdown, virtual visits (VVs) represented the first tangible action in favor of a home monitoring of chronic patients, obtaining positive results (11). A recent work published by Salzano et al. (12) was able to show in a cohort of 103 HF patients how a 24/7 audio and video management during the pandemic is able to decrease hospitalizations and mortality compared to a previously observed comparable population in which telemedical support was not present or available. While the feasibility and utility of such solutions for HF patients have been shown to be beneficial even before the pandemic (13), a lot of work is necessary to support a routine utilization. Even if advancements were made in terms of reimbursements, audio/video tools are not yet part of an organized widespread telemonitoring plan in all countries (11). Moreover, VVs require an important engagement by the medical doctors that many times does not match with the time available. Centralized hospitals dedicated only to telemedicine may solve such a problem. The Center for Cardiovascular Telemedicine in Charité Berlin is an example of how a centralized management of telemedicine information is able to act successfully on distant and rural territories (9).

Portable or wearable devices collecting vital parameters while involving the use of Web apps or smartphones are increasingly reliable, and they represent the next generation of solutions available for CHF patients (14). However, even if most research has shown the cost-effectiveness of such devices, regulatory authorities have slowed a full penetration of wearables in the medical market until further clinical evidence is available (15).

Another important aspect is technological, since the correct technology should match with the correct clinical indication. For example, for HF patients, a further step should be taken to move beyond the utilization of weight scales, known since many years to be poorly accurate in detecting body fluid congestion and body volume imbalances (14). While remote monitoring through implantable cardioverter defibrillators (ICDs) works really well for the detection of arrhythmias, the same cannot be said for the management of body fluids through intrathoracic impedance mainly due to the high risk of false positives that slowed the initial enthusiasm (16). Even if a lot of research is undergoing in new non-invasive technologies for the assessment of body fluids, their clinical value still needs to be demonstrated (14).

The complex clinical scenario offered by the COVID-19 pandemic should finally be the springboard for telemedicine. Telemedicine still presents challenges, such as the identification of the correct patients' populations in need, a variable that should always be addressed first. This has been nicely demonstrated in a recent randomized, multicenter, open-label telemedicine study by Galinier et al. (17), where patients at higher risk and the ones more socially isolated presented better clinical outcomes than more stable patients, showing how telemedicine may be more useful in such patients. Usability of technologies and increased adherence to the monitoring plan are some of the topics that need to be addressed to finally make telemedicine affordable and efficient for the post-pandemic healthcare system. On top of that, we believe that optimization of the healthcare organization and automatization of the management processes, meaning data collection, data interpretation, and clinical action toward the patients, need to proceed in a highly structured and fast path to be completely effective. This could be potentially achieved by departments or hospitals dedicated to telemedicine in conjunction with general practitioners. We believe that the introduction of working telemedicine programs needs to enter a novel stage, assigning specific duties and responsibilities to trained personnel. A collaboration between general practitioners and specialized centers is necessary mainly for medically underserved and rural areas. However, the roles need to be precisely defined to avoid confusion.

Both the Heart Failure Society of America and European Society of Cardiology strongly encourage the use of telemedicine for HF management during the COVID-19 outbreak (18). However, a functioning widespread system that allows the reimbursement of home monitoring solutions is still lacking (19). Germany is moving in an innovative direction with the so-called Digital Care Act, entitling all individuals covered by statutory health insurance to reimbursement for certain digital health applications (20). The chance of having a digital solution reimbursed encourages the hospitals to adopt novel telemedical solutions and produce proactively a much faster tangible outcome.

In Italy, during the COVID-19 outbreak, several patients experienced a poor outcome because they did not access to health system (21). Telemedicine owns nowadays the potential of delivering a better healthcare by empowering patients and by providing individualized healthcare, especially during a pandemic (22).

Yes, it is time for telemedicine. But, first, let us make telemedicine a matter of routine. Let us learn from our mistakes.

## Author Contributions

All authors listed have made a substantial, direct and intellectual contribution to the work, and approved it for publication.

## Conflict of Interest

AF and ELP are shareholders of the company BOCAhealthcare GmbH. The remaining authors declare that the research was conducted in the absence of any commercial or financial relationships that could be construed as a potential conflict of interest.
